# Learning to prescribe – pharmacists' experiences of supplementary prescribing training in England

**DOI:** 10.1186/1472-6920-8-57

**Published:** 2008-12-05

**Authors:** Richard J Cooper, Joanne Lymn, Claire Anderson, Anthony Avery, Paul Bissell, Louise Guillaume, Allen Hutchinson, Elizabeth Murphy, Julie Ratcliffe, Paul Ward

**Affiliations:** 1 Division of Social Research in Medicines and Health, School of Pharmacy, University of Nottingham, UK; 2 School of Nursing, University of Nottingham, UK; 3 School of Community Health Sciences, University of Nottingham, UK; 4 School of Health and Related Research, University of Sheffield, UK; 5 School of Sociology and Social Policy, University of Nottingham, UK; 6 University of South Australia, Adelaide, South Australia 5000, Australia; 7 Flinders University, Sturt Road, Bedford Park, Adelaide, South Australia

## Abstract

**Background:**

The introduction of non-medical prescribing for professions such as pharmacy and nursing in recent years offers additional responsibilities and opportunities but attendant training issues. In the UK and in contrast to some international models, becoming a non-medical prescriber involves the completion of an accredited training course offered by many higher education institutions, where the skills and knowledge necessary for prescribing are learnt. Aims: to explore pharmacists' perceptions and experiences of learning to prescribe on supplementary prescribing (SP) courses, particularly in relation to inter-professional learning, course content and subsequent use of prescribing in practice.

**Methods:**

A postal questionnaire survey was sent to all 808 SP registered pharmacists in England in April 2007, exploring demographic, training, prescribing, safety culture and general perceptions of SP.

**Results:**

After one follow-up, 411 (51%) of pharmacists responded. 82% agreed SP training was useful, 58% agreed courses provided appropriate knowledge and 62% agreed that the necessary prescribing skills were gained. Clinical examination, consultation skills training and practical experience with doctors were valued highly; pharmacology training and some aspects of course delivery were criticised. Mixed views on inter-professional learning were reported – insights into other professions being valued but knowledge and skills differences considered problematic. 67% believed SP and recent independent prescribing (IP) should be taught together, with more diagnostic training wanted; few pharmacists trained in IP, but many were training or intending to train. There was no association between pharmacists' attitudes towards prescribing training and when they undertook training between 2004 and 2007 but earlier cohorts were more likely to be using supplementary prescribing in practice.

**Conclusion:**

Pharmacists appeared to value their SP training and suggested improvements that could inform future courses. The benefits of inter-professional learning, however, may conflict with providing profession-specific training. SP training may be perceived to be an instrumental 'stepping stone' in pharmacists' professional project of gaining full IP status.

## Background

In recent years, UK pharmacists have experienced many changes to practice and increasingly undertaken a number of extended and enhanced services such as medicine use reviews, diagnostic testing and public health interventions. One of the most significant changes, however, has been the introduction of prescribing privileges for pharmacists, mirroring similar initiatives internationally [1]. Since 2003, UK pharmacists have been able to train and practice as prescribers – initially as supplementary and more recently also as independent prescribers [2-4]. There are currently around 1500 pharmacists registered as prescribers in the UK, the majority of whom have the supplementary prescribing (SP) qualification [5]. SP is a dependent form of prescribing, in which the care of a patient is shared between pharmacist and doctor, with the latter making the initial diagnosis and the former prescribing under the remit of a patient-specific clinical management plan – all with the patient's agreement. This may be contrasted with independent prescribing, which allows pharmacists to both diagnose and prescribe without the need for medical supervision [3]. In the UK, prescribing privileges are not limited to pharmacists but include nurses (who gained limited prescribing privileges in the late 1990s) and most recently, allied health professionals (AHPs) such as radiographers, podiatrists and physiotherapists, reflecting the UK government's aims of improving access to medicines for patients and better using the skills of healthcare professionals [2,6].

Despite the different forms of prescribing available and the range of professions to which they apply, a common feature is that an accredited prescribing training course at a higher education institution (HEI) must be successfully completed prior to practice. For pharmacists, this is in addition to their mandatory under-graduate pharmacy degree and this prescribing training has been referred to as the '*keystone' *[7] of the UK non-medical prescribing initiative. It may also be contrasted with non-medical prescribing models internationally – such as in many American states – where local assessment of competencies, rather than nationally recognised and accredited training, is more usual [1]. UK courses involve the equivalent of 26 day's taught study, and are part-time (lasting 3–6 months) with some HEIs also offering distance-learning options. The part-time nature of courses reflects the need for students to be actively engaged in a clinical area of practice where prescribing can be used. Indeed, it is another course requirement that prospective students have several years' clinical experiences (two for pharmacists, three for nurses and AHPs). In addition to the taught element, all SP courses require students to complete a further 12 days of learning in practice, where a designated medical practitioner (DMP) provides additional training, experience and guidance in relation to prescribing in practice. All courses are accredited by a relevant professional body and the Royal Pharmaceutical Society of Great Britain (RPSGB) – like the Nursing and Midwifery Council and Health Professions Council – stipulates indicative content and learning outcomes for SP courses.

UK prescribing courses have also been subject to change and, at the time of writing for example, pharmacist prescribing courses were undergoing accreditation to provide a combined IP and SP prescribing course, although interim top-up courses for pharmacist independent prescribing are available. A further trend is that of HEIs offering inter-professional courses, where prescribing neophytes from different professions are taught together. This reflects broader recognition within healthcare that providing combined training can provide a range of benefits, as stated in one of the common definitions of inter-professional learning wherein:

*"two or more professions learn from and about each other to improve collaboration and the quality of care."*[8]

Although pharmacist SP training is a relatively recent initiative, it has been explored in several studies [9], often involving early pharmacist training cohorts [10-12]. Overall, these studies reported that SP training was valued positively by pharmacists, in preparing them for their future prescribing roles [13], giving them confidence [7] and encouraging reflective learning [14]. However, aspects of courses such as the amount of course work to be completed and the limited time in which to undertake training (due to existing work commitments and the short duration of courses) were criticised. In addition, the inter-professional nature of some courses – where pharmacists trained with nurses and AHPs – received a mixed response, as did the pharmacology training provided. These latter concerns may be inter-related since it is recognised that nurses and pharmacists may have very different training needs [15] and that nurses, in particular, require significant pharmacology training [16-18] while pharmacists consider themselves competent in this area [6].

The aim in this paper is to explore the experiences and perceptions of pharmacists who completed SP training between 2003 and 2007. Including later cohorts allows us to consider the effect of changes to courses over time and subsequent use of prescribing in practice. Particular objectives were to explore possible tensions between increasingly inter-professional prescribing courses and specific elements of course content such as pharmacology training, and the relationship between SP and IP training.

## Methods

### Contextual Setting

As part of a larger, Department of Health funded evaluation of nurse and pharmacist SP in England, a national postal questionnaire of all 808 pharmacists qualified as supplementary prescribers in England was undertaken in April 2007. Contact data – names and addresses – for these pharmacists were provided by the RPSGB, using their records relating to the prescribing status of all pharmacists registered in the UK. A follow up letter and further copy of the questionnaire were sent to non-respondents after three weeks. All data returned were anonymised prior to publication and ethical approval for this national survey was obtained from a multi-centre research ethics committee (MREC).

### Questionnaire structure

The questionnaire was 10 pages long and consisted of 45 items divided into four sections: *'about you'*, *'training and support'*, *'your supplementary prescribing practice' *and *'supplementary prescribing and team working'*, with each section being a mixture of closed and open response questions; only the first two sections are relevant to this paper. The questionnaire was designed following a review of the literature relevant to SP [9] and discussions amongst the research team. The questionnaire was piloted on a small number of qualified supplementary prescribers – both pharmacists and nurses – and no problems with content or face validity were identified.

The first section of the questionnaire – *'about you'*- was designed to gather demographic data relating to pharmacists who had completed the training to become a supplementary prescriber and included fixed response questions in relation to gender, age, prior qualifications, area of practice, income and year of qualification. This section also contained open response questions in relation to whether respondents were currently prescribing, how long after registering with the RPSGB they started prescribing and their intention with regard to completing a conversion course in order to qualify as an independent prescriber.

The second section – *'training & support'*- asked respondents to rate their perception of their supplementary prescribing training on a five point scale ranging from 'strongly agree' to 'strongly disagree' in relation to four different statements:

- *'My supplementary prescribing training was useful'*

- *'My supplementary prescribing training provided the knowledge I needed in order to prescribe appropriately'*

- *'My supplementary prescribing training provided the skills I needed in order to prescribe appropriately'*

- *'My DMP fulfilled the role expected of them'*

Open response questions were also included to elicit respondents' views on the 'most useful' and 'least useful' aspects of SP training for pharmacists and areas for suggested improvements. A final question asked pharmacists for any additional comments on SP generally.

### Data Analysis

Quantitative data from returned questionnaires was analysed using SPSS (version 12), primarily to obtain descriptive (univariate) analyses, and Chi-square tests were used to analyse possible correlations between data. In relation to the open response questions, qualitative analysis was undertaken by one of the researchers, involving firstly open coding of all responses and subsequent refining of codes until all data were represented in coding categories. Simple tabulation of qualitative responses was also undertaken to estimate the frequency of responses in order to complement but not replace the qualitative analysis and help identify key themes [19].

## Results

### Demographics

Questionnaire responses were received from 411 pharmacists from an overall sample of 808 pharmacists, representing a 51% response rate after one follow-up reminder to non-respondents. General demographic results based upon completed questions indicated that the majority of pharmacist prescribers were female (75% 271/363) and that less than half (47% 193/411) were currently using supplementary prescribing. The respondents reflected SP training across the full period it had been taught at the time of the research, although those qualifying in 2007 were less represented as the survey was undertaken less than half way through that year (Table 1). There was a statistically significant association between current supplementary prescribing status and year of qualification as a supplementary prescriber, with 70.7% of pharmacists qualifying in 2004 reporting current prescribing, compared to 55.6% of those qualifying in 2005, and 44% of those qualifying in 2006 (χ^2 ^= 14.35, d.f. = 1, p = 0.002).

**Table 1:** Year of qualification as supplementary prescriber and current supplementary prescribing status

	When did you register by year?				
		
	2004	2005	2006	2007	Total
Currently using supplementary prescribing	53	60	53	6	172
Not currently using supplementary prescribing	22	48	67	9	146
Total	75	108	120	15	318

Only a minority of pharmacists had completed independent prescribing training, but almost all of the pharmacist respondents were either training or intended to train as independent prescribers (Table 2). There was statistically significant association between current supplementary prescribing status and independent prescribing status and intentions, with current supplementary prescribers being more likely to be already using, or training for, independent prescribing, than pharmacists not using supplementary prescribing (χ^2 ^= 42.804, d.f. = 3, p < 0.001). Pharmacists were also asked about their qualifications and, in addition to their undergraduate pharmacy degree, more than half (55% 225/411) reported having a postgraduate – often Masters level – qualification.

**Table 2:** Independent prescribing status and intentions

	In relation to independent prescribing – which best describes you?				
		
	Prescribing	Training to prescribe	Intending to train to prescribe	Not intending to train to prescribe	Total
Currently using supplementary prescribing	21	61	96	7	185
Not currently using supplementary prescribing	2	22	104	26	154
Total	23	83	200	33	339

### Attitudinal Questions

**Figure 1:**
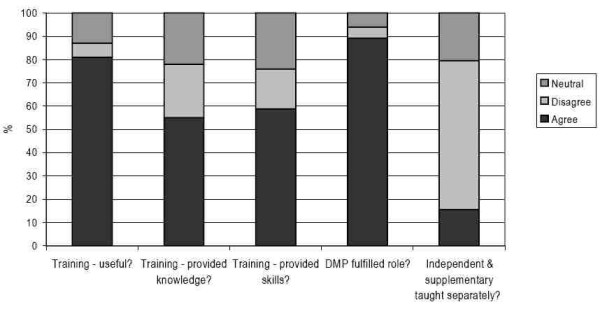
Pharmacists' responses to attitudinal questions.

SP training was perceived to be useful for most of the pharmacist respondents (82%, 288/351 agreed or strongly agreed) and doctors' roles in the supervision of pharmacists in the period of learning in practice was also highly rated, with 87% (305/353) of pharmacists agreeing or strongly agreeing that DMPs had fulfilled their roles (Figure 1). In contrast, pharmacists were less positive overall about whether training had provided them with relevant knowledge and skills – with 58% (205/354) agreeing/strongly agreeing that they had acquired the appropriate prescribing knowledge and 62% (217/353) agreeing/strongly agreeing that they had gained the skills they needed to prescribe. In addition, only a minority of pharmacist respondents (15% 53/35) agreed or strongly agreed that independent and supplementary prescribing should be taught as separate courses.

Possible associations between pharmacists' attitudes towards training and year of qualifying as a supplementary prescriber were undertaken using Chi-square analysis, but no statistically significant associations were identified across the four cohorts. Nor were there any significant associations between training attitudes and current supplementary prescribing status.

### Most useful aspects of training

In response to an open question, the most frequently cited useful aspects of training were the period of learning in practice and the involvement of their DMP, followed by gaining an understanding of the legal aspects of prescribing. Consultation and examination skills were also considered valuable parts of SP training, as was the contact they experienced with other healthcare professionals and their attendant support on inter-professional courses, where pharmacists were taught alongside nurses. Most pharmacists only listed one or two 'useful aspects' in their responses but others were more effusive:

*"Some practical training on diagnosis – e.g. chest percussion, blood pressure taking. Legal aspects – what can/cannot be prescribed. Good consultation skills when shadowing DMP." *P234

Overall, pharmacists' responses to the training questions were often succinct (even given the limitations of the questionnaire format) but a number of nuances in general themes did emerge. For example, within the broad theme of legal aspects of training, pharmacists found not only the legislation relating to SP useful, but also other legal concerns such as negligence, accountability and the responsibilities of the prescriber. It was apparent that pharmacists valued elements of training that gave insights into what subsequent prescribing practice would involve (such as when they observed and worked alongside doctors in the period of learning in practice), what additional skills would be needed (consultation, clinical and examination), what framework SP was possible within (in learning about law) and also recognising that becoming part of a team was necessary.

### Least useful aspect of training

Pharmacists also identified a number of negative aspects of their training, although despite being more varied in type these were much less frequently reported in total. By far the most frequently identified least useful aspect of SP training concerned pharmacology, with pharmacists noting that this aspect of SP courses was either '*too basic'*, taught them what they already knew or was a '*waste of time.'*

Some argued that more specific pharmacology relevant to clinical areas in which pharmacists intended to practice would have been useful, but it was also recognised that this would be difficult on courses designed not only for pharmacists from differing clinical areas but also different professions:

*"A lot of the course focused on pharmacology – very useful for the nurses on the course but not so useful for the pharmacists." *P39

Other negative comments about training concerned the format of the courses in terms of the amount of paperwork involved, the need to document and demonstrate competencies (including completing a portfolio), together with the time needed to actually attend university and the need the develop reflective practice skills. Some pharmacists also criticised the format of examination, such as the observed structured clinical examination, for example. Although not elaborated upon further by respondents, these often appeared to reflect a tension between on-going work commitments and not only attending the courses but also completing the directed reading and preparing for examinations. It was also apparent that there was not complete consistency amongst the pharmacist sample and, for example, although law was cited as the most useful element for many, other considered it the least useful aspect of training. Similarly, whilst many found gaining clinical skills the most useful, others found this the least helpful part, especially as some had been taught what they considered to be skills that were irrelevant to their clinical area, such as performing venepuncture or using an otoscope, for example. Others recognised that this was understandable given the scope of the course and appreciated the difficulty in addressing such aspects of training in anything more that a cursory way.

There was also disagreement over issues such as the development of inter-professional courses which taught pharmacists alongside nurse and/or AHPs. As noted, some considered such course advantageous in facilitating networking and support, offering an "*appreciation of other health care professionals' views and perceptions towards patient care." *Others considered inter-professional courses disadvantageous, in trying to teach professionals with different existing skills and knowledge, such as pharmacology.

*"Because it was a generic course dominated by nurses, there were always sessions not totally appropriate but a more specific course would mean you lose out on other professionals experiences which are invaluable." *P106

Of particular interest, however, was several pharmacists' comment that there were no least useful aspects of training, implying that the course content was relevant and useful.

### Suggested improvements to training

Related to the above point was the fact that several pharmacists noted that nothing needed to be added to SP courses. However, other pharmacists did offer suggestions as to what should be covered and although some 34 distinct issues were raised by pharmacists, those most frequently identified all involved issues that pharmacists also found useful in their course, namely examination, consultation and clinical skills. In addition, training that involved specific clinical areas was suggested by several pharmacists, as well as training in diagnosis. Although diagnosis does not form part of the core curriculum for SP, its popularity as a suggested improvement for SP training appeared to reflect pharmacists' desire to understand, and be involved more fully with, the treatment of patients:

*"The fundamental issue I had was that as a SP you are not taught about diagnosis, which when other disease states occur in a patient – you do not have the full extent of training as a medic hence [...] you cannot do the complete job for the patient" *P361

*"More diagnostics to help understand when to refer back – would have given me more confidence." *P355

Although such course suggestions reflected pharmacists' desire to improve their subsequent prescribing practice, diagnosis training may also be viewed as an important and distinguishing feature of IP, and further implications of pharmacists' references to diagnosis training will be considered next and in the discussion.

### Other Comments

Pharmacists were also invited to add further comments about SP generally and several comments relevant to training were reported – particular references being made to the value of pharmacists' clinical experience. One pharmacist used such experience to argue against planned proposals to teach prescribing on undergraduate pharmacy, noting that SP training and practice resulted in increased responsibilities, which newly qualified pharmacists would find difficult. Another pharmacist noted:

*"Having eight year's experience in primary care and completed a PG Dip [post-graduate diploma] in Prescribing Sciences, much of the formal SP course was irrelevant and a duplication of knowledge." *P10

Related to the results of pharmacists' intentions to train and prescribe as independent prescribers, many pharmacists commented that SP and the associated training were a '*stepping stone' *to IP status and training. SP training appeared to be viewed instrumentally, in preparing pharmacists for a transitional stage in their prescribing, although such training was not viewed negatively by most pharmacists:

*"I have now qualified as an IP. I find that the training and subsequent year I spent in SP an entirely useful foundation for my development to IP." *P155

## Discussion

Pharmacists appeared to value their SP training overall, identifying both positive and negative aspects and suggesting improvements, and revealing aspirations about IP training and practice. The only significant association in relation to when pharmacists undertook courses was that early cohorts were more likely to be currently using SP in practice. Although inferences about the influence of actual courses are not possible, a practical explanation may be that earlier cohorts will have had more time and opportunities to begin prescribing compared to later cohorts. There was no significant association between training cohorts and attitudes towards courses although all cohorts reported some concerns about SP training that have emerged in earlier studies [9], such as dissatisfaction with being taught basic pharmacology and mixed responses to inter-professional training. That these concerns are emerging even amongst later cohorts is interesting since SP courses and content have changed and continued dissatisfaction with pharmacology, for example, was identified despite changes to indicative course content guidance offered by the RPSGB, which no longer involves an:*'update on relevant aspects of basic and applied therapeutics [and] clinical pharmacology' *[20]. The use of approved prior learning (APL) – where some course components may be omitted if evidence of previous training and competency can be shown – may be relevant. However, APL cannot currently exempt pharmacists from whole sections of courses and although individual HEIs may allow pharmacists to negotiate exemption from some *taught *elements (such as numeracy which nurses must undertake), pharmacists must still sit and pass *every *course exam.

It was apparent that pharmacists' perceived themselves to be competent in their pharmacological knowledge. This has also been identified in previous research [6,21] and may be contrasted with empirical research [16] and lecturers' perceptions [17,18] about *nurses' *pharmacological competency and training needs. Pharmacists' undergraduate pharmacology training and the number of post-graduate qualifications identified may inform this perceived competence but concerns remain. Firstly, if pharmacists consider SP course pharmacology content to be too easy or basic then does this mean that nurses and AHPs might be receiving pharmacology training that is also too basic? Secondly, pharmacists' pharmacological knowledge may still need to be assessed formally in a course exam, to ensure a minimum competency, and empirical research is needed to confirm pharmacists' pharmacological competency.

Pharmacists' desire to learn more about diagnosis, consultation and clinical examination skills is revealing since although some clinical examination and consultation skills are required on SP courses, pharmacists wanted both more of these skills and also training in *diagnosis*, which is not directly relevant to SP. This may be related to most pharmacists' intentions of becoming independent prescribers and belief that independent and supplementary training be taught together. Pharmacists' references to SP being a *'stepping stone' *to IP were telling and SP training appeared not only to prepare pharmacists for initial prescribing but also represented the first part of a larger professional project towards independent prescribing.

The findings of this study suggest that inter-professional learning may be problematic despite the benefits offered. Challenging the traditional approaches to health care training that were distinct and proceeded along very *'discrete occupational lines' *is recognised as an important project [22]. Benefits of increased understanding of different professions and improved communication and collaboration are all laudable aims that could have benefits not only in training but also in subsequent practice, reflecting a more fundamental socialization process that can occur in healthcare training [8,22]. However, the need to provide different professions with potentially different sets of skills may be difficult to accommodate in practice. Pharmacology training and numeracy exams for nurses illustrate these quite different training needs.

This study offers suggestions as to possible changes to prescribing course content. Obvious points are that pharmacology content should be reduced or made specific to pharmacists' clinical areas, that inter-professional courses be offered only if they can avoid teaching of existing knowledge and that more diagnostic, consultation and clinical examination skills should be offered, preferably on an integrated SP/IP course. Other possibilities are to include more practical aspects of training, as this was especially valued by pharmacists, possibly by involving practicing non-medical prescribers in courses and most importantly increasing the period spent with a doctor in practice, which pharmacists' particularly valued. In the UK, doctors are presently not remunerated for their roles as DMPs and this may need to be addressed, to both encourage more doctors to participate and also to ensure that their time and skills are recognised. If the role of the DMP is to be increased, however, more attention may also need to be given to doctors' understanding and awareness of SP, since research suggests that this may be lacking at present [9]. Finally in relation to course changes, giving more consideration to pharmacists' existing workload or making courses longer or more flexible may also be needed.

Finally, this study may inform proposed changes to pharmacist SP training. One is that all pharmacist courses will eventually offer a combined SP and IP course, bringing pharmacist training more in line with that of nurses, where a single extended, independent and supplementary prescribing qualification is now offered. The findings of this study indicate that this process would be welcomed by pharmacists, in allowing them to obtain diagnostic and clinical skills and attain their ultimate goal of IP status. A further issue concerns the integration of prescribing training into the undergraduate curriculum, as has been proposed [23] and this study raises questions about such a proposal. In particular, how will undergraduate students' lack of clinical experience affect the success of such courses and will it be possible to incorporate the period of learning in practice and time with a DMP that pharmacists so valued into an undergraduate course? Such concerns have emerged in other research [9] but further research will be needed to assess these developments.

In terms of study limitations, the response rate means that it may not be possible to generalise from this sample to all RPSGB registered pharmacists, or those in other parts of the UK. Due to study time limitations, it was not possible to analyse non-respondents. The limitations of a questionnaire format must also be recognized and, for example, the data obtained from the open response questions was often succinct and pharmacists did not describe or articulate their experiences and perceptions in the same detail that, for example, qualitative interviews might have permitted. Finally, the survey included pharmacists from early cohorts and so the results do not necessarily reflect pharmacists' views about only the most recent SP courses.

## Conclusion

The pharmacists in this survey appeared to value their SP training in preparing them for practice. However, aspects of courses such as the pharmacology content continue to be problematic and this may be heightened as an increasing number of HEIs offer inter-professional courses, despite possible APL use and proposals for integrating prescribing into undergraduate curricula. Several improvements to prescribing courses may be not only desirable but also necessary. Pharmacists' views about IP and more diagnostic training appear to reflect a view that SP and SP training are but a 'stepping stone' to fully independent prescribing.

## Competing interests

The authors declare that they have no competing interests.

## Authors' contributions

PB and PW conceived of the study, RJC and LG participated in the collection and analysis of data, RJC drafted the manuscript and all the authors contributed to the design of the study and read and approved the final manuscript.

## Pre-publication history

The pre-publication history for this paper can be accessed here:


